# Pericoronitis: A clinical and epidemiological study in greek military recruits

**DOI:** 10.4317/jced.55383

**Published:** 2019-02-01

**Authors:** Thomai Katsarou, Andreas Kapsalas, Christina Souliou, Theodoros Stefaniotis, Demos Kalyvas

**Affiliations:** 1 DDS, MSc. Department of Oral & Maxillofacial Surgery. Dental School, University of Athens, Greece; 2 DDS. Department of Oral & Maxillofacial Surgery. Dental School, University of Athens, Greece; 3 MD, DDS. Department of Oral & Maxillofacial Surgery. Dental School, University of Athens, Greece; 4 DDS, Dr. Dent. Department of Oral & Maxillofacial Surgery. Dental School, University of Athens, Greece

## Abstract

**Background:**

This paper presents a statistical analysis of epidemiological, clinical and radiographical characteristics of third molar-related pericoronitis.

**Material and Methods:**

650 conscripts of the First Training Division of Conscript Soldiers of 2005 in Greece were recruited for the study. Each conscript was given a questionnaire and underwent a clinical test and a radiographic examination. The tested variables included the conscripts’ personal information, oral hygiene parameters along with the radiographic angulation of the third molar, the level of impaction and their classification in relation to the edge of the mandible.

**Results:**

The prevalence of pericoronitis was found to be 4.92%. The group of patients between 20 and 25 years old dominated in a percentage of 72.41%.

**Conclusions:**

The use of mouthwash along with the adequate frequency of teeth-brushing appeared to be related to a statistically significant decrease of the disease. Vertical impacted molars are more likely to present pericoronitis at a rate of 61.11%; plane A and the impacted teeth that are positioned to the front edge of the mandible according to class II, have a higher rate of prevalence. Finally, a brief literature review in comparison to our study is also presented.

** Key words:**Third-molar-related pericoronitis, impacted wisdom teeth, prevalence, epidemiological study, Greece.

## Introduction

Pericoronitis, also known as operculitis, is defined as an inflammation of the soft tissue surrounding the crown of an impacted or semi-impacted tooth (1). The soft tissue covering a partially erupted tooth is known as an operculum in clinical terms. Pericoronitis mainly refers to the inflammation of the soft tissue surrounding third molars as it is quite infrequent to occur elsewhere (2).

The condition is most commonly seen in late adolescence or early adulthood life and its development is attributed to a variety of factors. The oral microflora may develop a pathologic potential under low immune resistance (stress, viral recovery) and assist to the presentation of symptoms (3). Furthermore, a developed operculum surrounding the teeth encourages bacterial plaque retention in between, along with the chewing trauma caused by the antagonist; all the above are considered to be aggravating factors of pericoronitis.

Various studies have been analyzing the special types of microbes mainly causing the condition and have concluded to Streptococci milleri as one of the most predominant types. An interesting result is considered to prove that necrotic ulcerative gingivitis and pericoronitis share similar responsible microbe strains (4).

Symptoms may include pain, swelling and tenderness along with difficulty in mouth opening and discomfort in swallowing. Lymphadenitis of the associated lymph nodes, fever, malaise, unpleasant breath/taste accompanied by purulent exudates of the operculum revealed upon palpation, and loss of appetite may additionally be present (5). The frequency and intensity of these symptoms are closely related to the clinical category of the disease. Three categories are clinically and diagnostically recognized, namely acute, sub-acute and chronic pericoronitis, each one forming a different symptomatologic profile of the patient. Acute pericoronitis is characterized by limited mouth opening and more severe symptomatology, sub-acute pericoronitis follows a similar pattern in a lower intensity and without any report of mouth opening discomfort, and chronic pericoronitis refers to patients describing a short-lasting low-grade pain without significant symptomatology (6,7). Moreover, several studies have proved that the disease may affect patients’ quality of life with compromising and adverse outcomes (1).

The aim of this study was to determine the epidemiological and clinical characteristics of pericoronitis and investigate factors which can affect the prevalence of pericoronitis, such as demographic and social factors, along with clinical and radiographic determinants.

## Material and Methods

The study was carried out in a sample of 650 conscripts of the First Training Division of Conscript Soldiers of 2005, who were recruited in the transmission center.

All soldiers completed a question form and underwent a clinical test and a radiographic examination. Additionally, all data was statistically analyzed and the prevalence of the condition was related to the different parameters included in the study.

A special question form was designed, inquiring personal information like age, socioeconomic status and medical history, and including questions about oral hygiene habits and the findings of the radiograph and clinical examinations. All participants were categorized into three groups according to their age. The first group included participants under 20 years old, the second group included the ones between 20-25 years old and the third group referred to participants between 26 and 30 years old. The socioeconomic status was identified through questions regarding the place of residence, depending on urban, sub-urban or rural location, the participants’ profession and the profession of their father, along with the educational level. The medical record concerned systematic or other diseases at the time of study, in addition to medication treatment.

The clinical examination was performed by dentists using the diagnosis of pericoronitis as the main criterion. In cases where pericoronitis was confirmed, the clinician recorded the operculum size (minor, medium, wide), the responsible tooth and the clinical classification of the disease (acute, sub-acute, chronic). Furthermore, the oral hygiene level was examined via the plaque index (PLI), the papilla bleeding index (PBI), the Decayed-Missing-Filled index (DMFI) and via the recording of mucosal health habits, such as brushing frequency and the possible use of mouthwash. Finally, questions concerning smoking were integrated in the questionnaire and the participants were classified into light smokers if fewer than 10 cigarettes were consumed per day, moderate smokers if the number of cigarettes was 10-20 and heavy smokers if the number of cigarettes exceeded 20.

Radiographic findings were recorded in our study via panoramic x-rays. The angulation of third molars was determined and they were grouped into vertical, mesioangular, distoangular and horizontal, according to the angle they form with the main axis of the adjacent second molar. The position of the third molars in association with the occlusal plane was also noted according to the Pell and Gregory’s Classification: they were classified into the ones of level A, in which case the crown is in a similar height to the cervical of the second molar and level B, with the crown being lower than the cervical of the second molar. The final radiographic aspect was the classification of third molars in relation to the edge of the mandible, in Classes I, II and III.

Data concerning the different aspects related to pericoronitis was analyzed using regression analysis (one-way Anova, chi-square test, pearson correlation) with SPSS 17 (SPSS Inc. Chicago IL) with the level of significance set at *P* <0.05.

## Results

**Figure 1:**
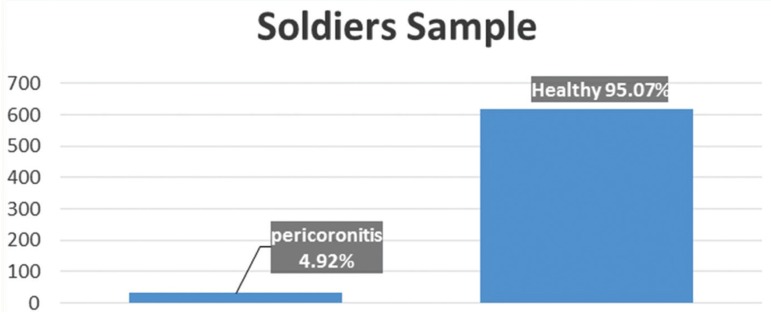
Participants suffering from pericoronitis.

Pericoronitis was clinically diagnosed to 32 participants out of 650 of the original sample (Fig. 1). Table 1 presents the socioeconomic status of 28 patients with diagnosed pericoronitis; 4 of them were excluded as the questionnaire data was insufficient. The group of patients between 20 and 25 years old dominated, in a percentage of 72.41% ( Table 1). The prevalence of pericoronitis was found to be statistically significant in urban population in a rate of 79.3%.

**Table 1:**
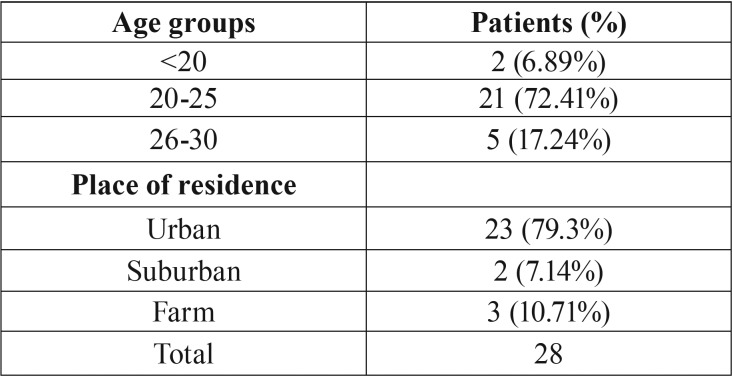
Demographic profile of patients. Relationship between age and disease and disease and residence. For 4 patients the data was insufficient.

 Table 2 presents the correlation between the participants’ oral hygiene habits and the prevalence of pericoronitis. The use of mouthwash along with adequate frequency of teeth-brushing appeared to be related to a statistically significant decrease of the disease ( Table 2). Nevertheless, the PLI (plaque index) proved to be statistically insignificant, as pericoronitis appeared in 31.03% of the recruits whose PLI rate was 75 or above (worst mucosal hygiene). All those variables are highly correlated due to the great influence of brushing frequency and mouthwash use on PLI. Consequently, the confirmation of the above results requires a study of higher statistical power. The final result concerned smokers that suffered from the disease at a rate of 72.41% ( Table 3), whereas pericoronitis was not affected by the number of cigarettes consumed per day.

**Table 2:**
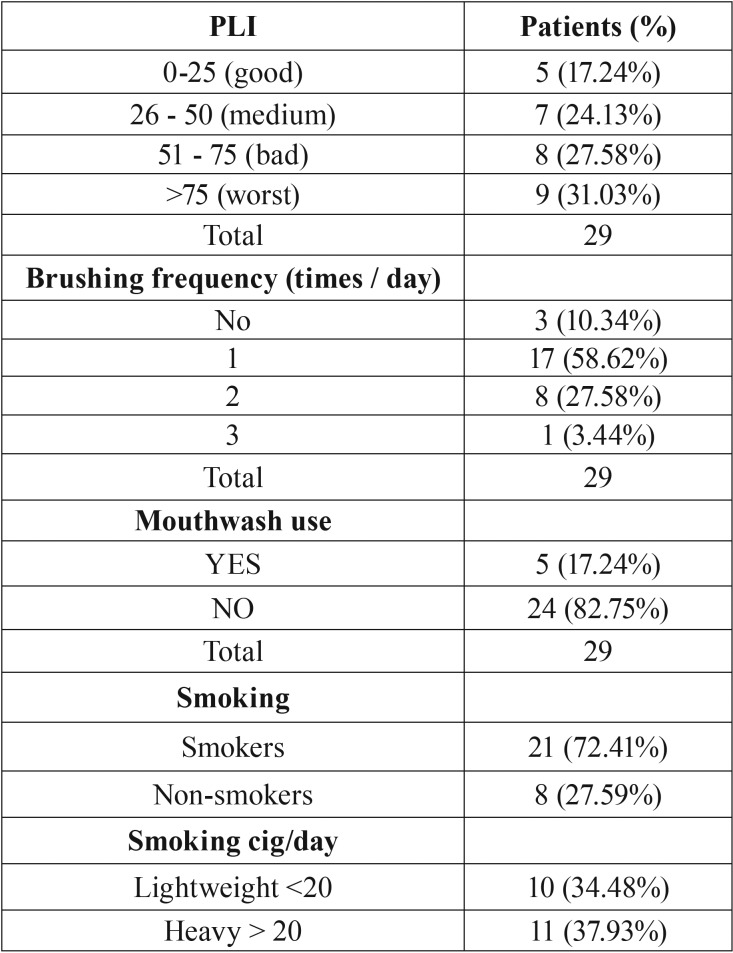
Oral hygiene and smoking habits of patients. Relationship disease; plaque index, disease; brushing frequency, disease- mouthwash use, disease; smoking heavily. The data is insufficient for three patients regarding the first three parameters and for 11 patients regarding the last parameter.

**Table 3:**
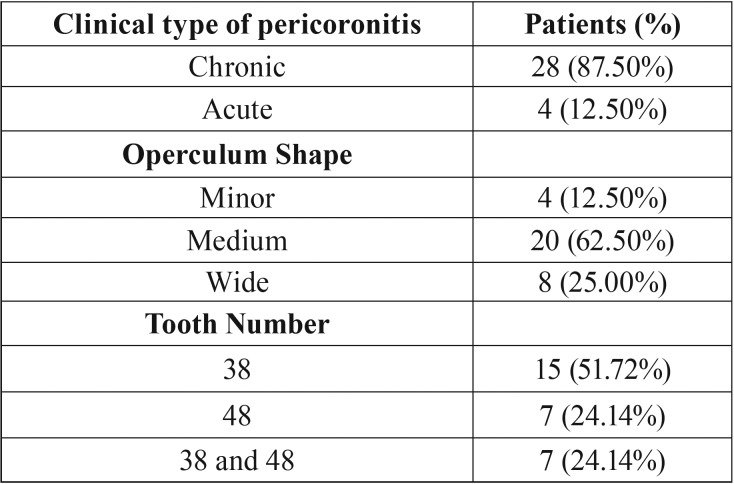
Clinical Examination Results. The prevalence of the clinical type of pericoronitis, the operculum shape and the semi-impacted tooth number.

The outcomes of the clinical examination are summarized in  Table 4. As expected, chronic pericoronitis was found to be the most common type of pericoronitis (87.50%) due to the severe, painful symptoms of the acute type that force patients to seek treatment ( Table 4). Medium size operculum was presented at a rate of 62.50% as the most responsible for plaque accumulation, and the lower left third molar is associated with the disease in more than half of the cases ( Table 4).

**Table 4:**
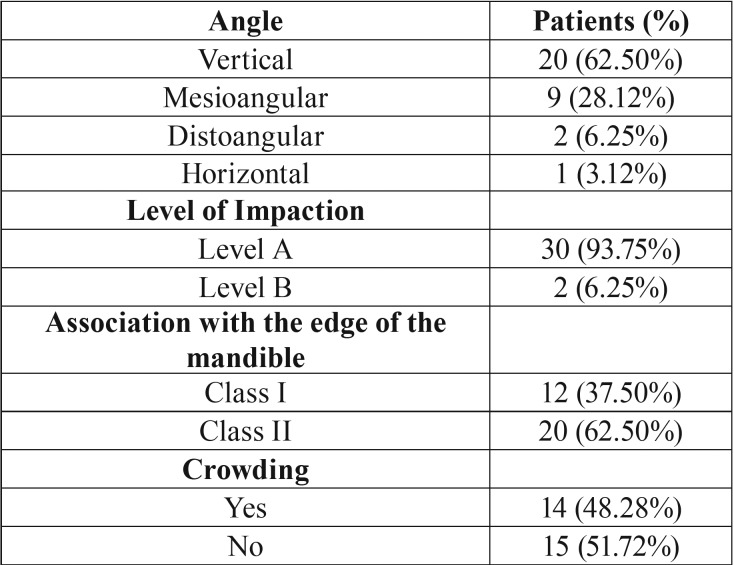
Radiographic examination results. The correlation of pericoronitis with angle, level of impaction, the edge of the mandible edge and crowding.

Through the study of panoramic radiographs, we obtained the evidence summarized in  Table 4. Vertical impacted molars are more likely to be affected by pericoronitis at a rate of 62.50%. This result may be anatomically explained by the fact that vertical impacted third molars come into great contact with the operculum and, consequently, the most severe mechanical trauma is caused. In addition, level A third molars -as more susceptible to additional trauma- were found to be inflamed in the majority of the participants (93.75%). Finally, the association of the impacted molars to the front edge of the ascending branch of the mandible was found to be statistically significant in the case of Class II due to insufficient space, because of which the tooth has few self-cleaning possibilities. As far as teeth-crowding is concerned, the evidence did not yield statistically reliable results.

## Discussion

Clinical trials have indicated that pericoronitis is one of the most significant reasons underlying the extraction of the third molars of the mandible (1). In an earlier study, Chestnutt *et al.* (1999) conclude that out of 613 patients of Scotland population, 5.5% of the examined teeth were extracted due to the disease (8). A later study of Adeyemo *et al.* (2008), in a sample of 1763 patients, states that the acute type of pericoronitis was the main reason behind 9.2% of teeth extractions, while the chronic type was the main reason behind 26.3% of the extractions (9,10). In the present study, the prevalence of pericoronitis was confirmed at a rate of 4.92% of the cases, whereas the chronic type dominated at a rate of 83.33% of the cases, a result which is also supported by a previous study of Lee *et al.* (11).

The particular army life, regarding the schedule, the hygiene and nutrition can also affect the prevalence of pericoronitis. The present study was carried out in an environment where the presence of stress is assumed as a predisposing factor, since participants consisted of newly arrived army recruits. In 2003, Batanieh *et al.* confirmed that stress as well as upper respiratory tract infection are predisposing factors of pericoronitis; indeed, stress was confirmed at a rate of 22% in a sample of 2151 patients (5).

The medical history of the participants of our study was not available and for this reason medical history cannot be under investigation as an affecting parameter.

As far as age is concerned, in the present research the majority of the participants belonged to the age group of 21-25 years old, which is a result confirmed by most previous clinical trials (11-13,5), although some of them estimated a slightly younger age range (19 to 24 years old) as the mean age of the disease (9,10).

The radiographic characteristics of wisdom teeth are indicated to be crucial aspects for the development of pericoronitis. Comparing to previous studies, Halverson-Anderson *et al.* (12) stated that teeth at highest risk of pericoronitis are the vertical third molars in contact with the second molar (II CLASS) at or above the occlusal plane (A- level) . Those results are in agreement with the study of Lee *et al.* (1989) (11). In a later study, Yamalik *et al.* (2008) have proposed the same results at a rate of 51% for vertical third molars and 57% for third molars of Class II (10). Finally, the study of Hazza’a AM *et al.* (2009) also agrees with the above results but in a percentage, which is not statistically significant (13).

Most studies that investigated pericoronitis indicate that the third molars of the mandible are more likely to be affected. Our study presented lower left third molars to be more susceptible to the disease in agreement with the study of Ayanbadejo *et al.* (14), which reported that lower left third molars (45,3%) were more affected than lower right third molars (37.1%) or than a combination of both lower third molars (17.7%) (15). Another study of Folayan *et al.* (2013) proved that pericoronitis may be present in non-third molar teeth at a minimum rate of 0.63%, with the lower left permanent second molars being more susceptible to the condition. The study was limited to children under 15 years old (2).

Magraw *et al.* 2015 reported that pericoronitis can affect patients’ total well-being, causing several troubles, including chewing, talking, mouth opening, sleeping, taking part in social life, participating in sports (16).

## Conclusions

The prevalence of pericoronitis in our study is 4.92%. The mean age of appearance is estimated between 20-25 years old and urban population is reported to be more susceptible to the disease. Oral hygiene habits and PLI were found to be marginally significant. Smokers are more susceptible to pericoronitis, but smoking frequency is not associated with the disease. The chronic type of pericoronitis is the most common one. The medium size of the operculum is more frequent and tooth #38 is more likely to get the disease. The vertical angle of occlusion, plane A and the impacted teeth that are positioned to the front edge of the mandible according to class II, have a higher rate of prevalence. Regarding teeth crowding, a statistically significant difference is not confirmed. Future studies including larger groups of participants should be necessarily conducted in order to establish additional possible correlations.
